# Magnetic Particle Self-Assembly at Functionalized Interfaces

**DOI:** 10.1021/acs.langmuir.0c03235

**Published:** 2021-04-02

**Authors:** Apurve Saini, Katharina Theis-Bröhl, Alexandros Koutsioubas, Kathryn L. Krycka, Julie A. Borchers, Max Wolff

**Affiliations:** † Department for Physics and Astronomy, Uppsala University, Uppsala, Sweden; ‡ University of Applied Sciences, Bremerhaven, Germany; § Jülich Centre for Neutron Science JCNS at Heinz Maier-Leibnitz Zentrum (MLZ), Forschungszentrum Jülich GmbH, Lichtenbergstraßze 1, 85748 Garching, Germany; ∥ NIST Center for Neutron Research, Gaithersburg, Maryland 20899-6102, United States

## Abstract

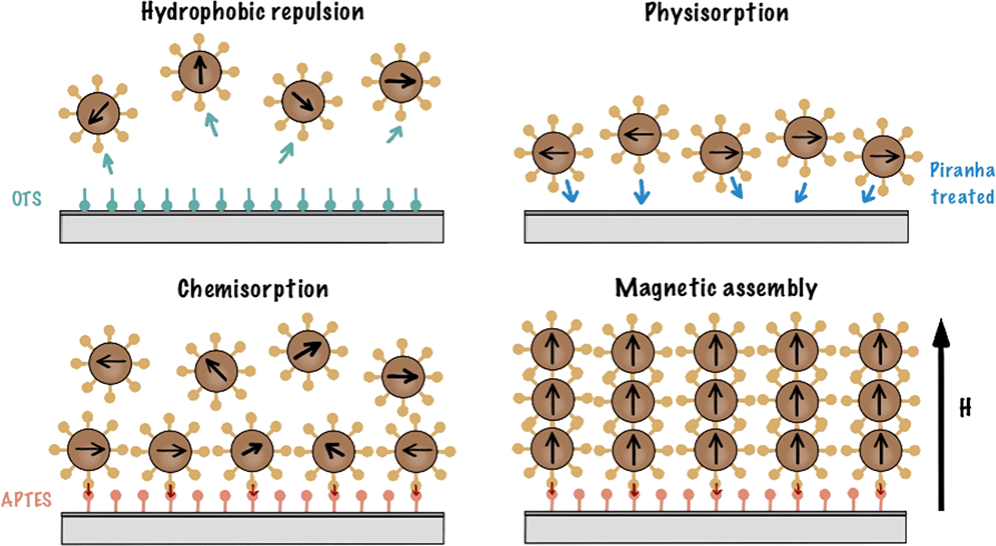

We study the assembly of magnetite nanoparticles in water-based ferrofluids in wetting layers close to silicon substrates with different functionalization without and with an out-of-plane magnetic field. For particles of nominal sizes 5, 15, and 25 nm, we extract density profiles from neutron reflectivity measurements. We show that self-assembly is only promoted by a magnetic field if a seed layer is formed at the silicon substrate. Such a layer can be formed by chemisorption of activated *N*-hydroxysuccinimide ester-coated nanoparticles at a (3-aminopropyl)triethoxysilane functionalized surface. Less dense packing is reported for physisorption of the same particles at a piranha-treated (strongly hydrophilic) silicon wafer, and no wetting layer is found for a self-assembled monolayer of octadecyltrichlorosilane (strongly hydrophobic) at the interface. We show that once the seed layer is formed and under an out-of-plane magnetic field further wetting layers assemble. These layers become denser with time, larger magnetic fields, higher particle concentrations, and larger moment of the nanoparticles.

## Introduction

The formation of ordered nanoparticle (NP) structures can be realized by self-assembly. A rich diversity of structures can be formed as result of the tunable interactions such as steric, electrostatic, and/or magnetic.^1,2^ However, only a detailed understanding of the underlying principles will allow the fabrication of tailor-designed smart/stimuli-responsive synthetic materials, resulting from the fact that self-assembled nanostructures can show remarkable collective properties that are different from their individual counterparts.^3,4^

One interesting class of materials in this context are magnetic nanoparticles (NPs) dispersed in a solvent, since they can self-assemble and are responsive to external stimuli (magnetic field). This enables a range of applications such as magnetic sealing and magnetic memory or in biomedicine.^5−7^ These applications make use of the ability of colloidal magnetic NPs to form structures such as linear or branched chains, clusters, or rings in an applied magnetic field.^8−10^ Similar applications are considered for thin films of magnetic NPs with the additional advantage that the self-assembling structure can be prepatterned and then grown from a substrate. Even without an applied field, self-assembly can take place due to the magnetic dipole interactions of single domain particles.^11,12^ Neutron reflectivity (NR) measurements are a unique tool (high penetration into silicon, sensitivity to magnetic induction, and isotope contrast variation) to extract information about the self-assembly of magnetic particles at solid substrates. From the specularly reflected intensity, nuclear and magnetic density profiles across interfaces can be extracted with high precision.^13−15^

Following along this line, Vorobiev et al.^16^ reported a dense wetting double layer of ferrofluid (FF) (9 vol % of 5.5 nm sized Fe_3_O_4_ particles in D_2_O) forming at a horizontal Si/SiO_2_ surface after 1 h. A DC magnetic field of 10 mT applied parallel to the solid substrate resulted in short-range ordering in the particle layers whereas a field applied perpendicular to the substrate resulted in long-range ordering. Moreover, it was found that the particle layering gradually develops over 48 h with long-range ordering (30 layers) at the FF–SiO_2_ interface. Recently, Kubovcikova et al.^17^ studied the correlation of the adsorption of NPs from aqueous magnetic fluids on a crystalline silicon surface with the bulk structure extracted from small-angle neutron scattering (SANS). Gapon et al.^18^ used two kinds of FFs: first, FFs with MNPs coated by a double layer of sodium oleate and, second, a FF with cobalt ferrite NPs stabilized by lauric acid/sodium *n*-dodecyl sulfate. The authors reported the formation of just one single adsorption layer for both FFs.

In a previous study, we have investigated the assembly of 11 nm Fe_3_O_4_ particles dispersed in D_2_O/H_2_O at a SiO_2_/Si surface under the influence of magnetic field and shear in a vertical sample geometry.^19^ This geometry has the advantage that sedimentation is avoided. The slightly elliptical particles oriented in an in-plane (field in the plane of the substrate/FF interface) magnetic field with their long axis along the field direction. Under shear, a dense wetting layering at the surface and a depleted region toward the moving FF were found. This assembly can be improved by chemical anchoring at (3-aminopropyl)triethoxysilane (APTES) functionalized Si substrates.^20^ In a more recent NR study, we show that FF NPs can be firmly attached to magnetic substrates.^21^ Dense and stable layers were found for dilute (0.15 vol %) solutions of 5, 15, and 25 nm sized Fe_3_O_4_ particles in D_2_O/H_2_O at an amorphous ferrimagnetic film (Tb_15_Co_85_) deposited onto a Si crystal.^21^ We show that once the first layer is formed, further NP assembly takes place as a result of the dipolar magnetic interaction and stray fields from the substrate.

Here we present a detailed investigation of the assembly of magnetic FF NPs at solid substrates with different functionalization of the substrate. We show that layers self-assemble if two conditions are fulfilled. First, a wetting/seed layer forms resulting from the affinity of the NPs shells and substrate coating. Second, once this layer has formed, the long-range dipolar magnetic interaction triggers the assembly of further layers.

## Sample

**Figure 1:**
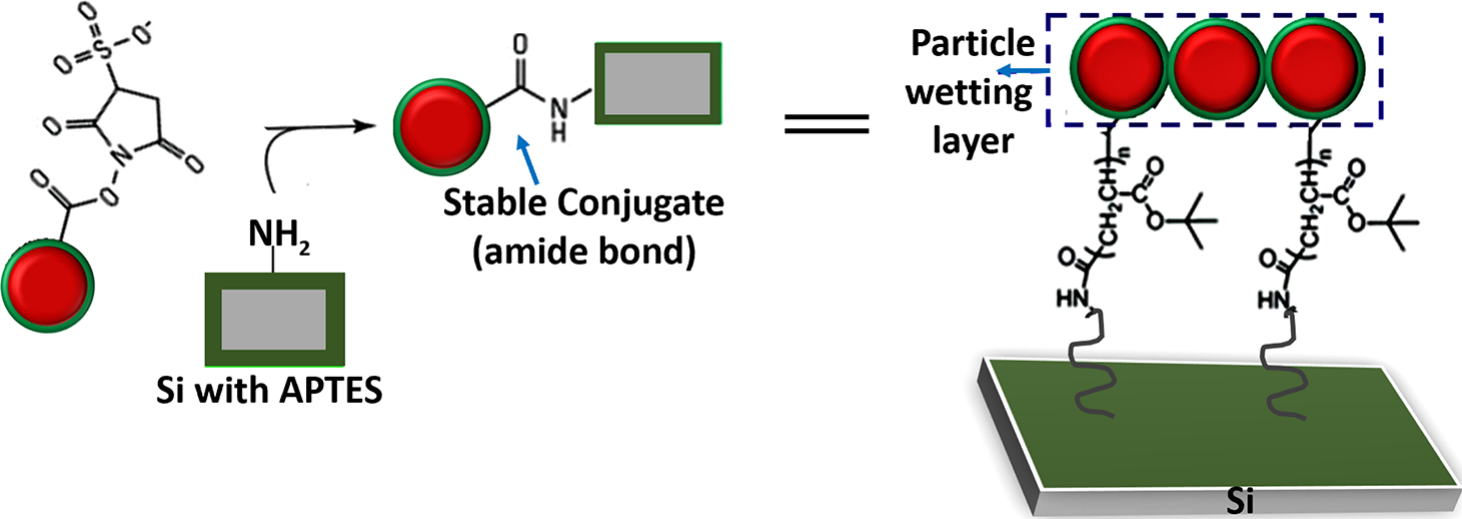
Anchoring of magnetic particles at functionalized surfaces. The NHS ester complex attaches to APTES (nucleophile).

NPs are often coated with oleic acid as a surfactant to make them stable in solution. However, this coating is not compatible with water as solvent due to the terminal methyl groups. An alternative coating, which makes the particles stable in water, is an activated *N*-hydroxysuccinimide (NHS) functionalization of the NPs. This coating has the additional advantage that bioconjugation chemistry^22−24^ leads to these NPs readily coupling, for example, with APTES-coated substrates^19^ through the highly stable and covalent amide linkages (−CONH bonding) between amine-terminated silicon surfaces and reactive carboxyl groups on the NP (see Figure 1).

To investigate the interaction between the MNP coating and different substrate coatings, NHS functionalized dried NPs were commercially obtained from Sigma-Aldrich.^a^ The size and shape of the NPs were verified by transmission electron microscopy (TEM) and confirmed by X-ray powder diffraction (XRD).^21^ The spherical nanocrystals show a narrow size distribution with average diameters of 4.1(5) nm (FF5), 14.9(6) nm (FF15), and 22.2(11) nm (FF25)^b^ and single crystal structure.^25,26^ Hysteresis loops extracted by superconducting quantum interference device (SQUID) magnetometry on dried powder samples show that the particles have negligible coercivity and a size-dependent saturation magnetization (*M*_s_) of 38.0, 50.8, and 72.3 emu/g for the samples FF5, FF15, and FF25, respectively, at room temperature (300 K). For the data see the Supporting Information. All values are lower than that of bulk magnetite (92 emu/g).^21^ SANS measurements were performed at the NGB30m SANS instrument at the NIST Center for Neutron Research (NCNR). The NPs were diluted in a mixture of 85% D_2_O and 15% H_2_O, for good contrast for neutrons, and contained in titanium sample cells with quartz windows with a separation of 2 mm. The sample–detector distances were 1, 4, and 13 m. To increase the *Q*-range, the detector was offset horizontally by 25 cm for the 1 m configuration. The wavelength was λ = 6 Å. For the low *Q* regime in the 13 m configuration, refractive neutron lenses were used. The wavelength spread was 13.8% (FWHM) and defined by the velocity selector in all configurations. Fits to the reduced data assume a power exponent together with polydispersed core/shell spherical NPs for each sample, and the results are tabulated in Table 1. A more detailed description of the above characterizations of the NPs is presented in ref (21), and the SANS and magnetometry data are reproduced in the Supporting Information. In ref (21) we studied the self-assembly of the same NPs at magnetically template substrates.

**Table 1:** Results of Fits to the SANS Data Assuming a Linear Combination of a Power Law and Core/Shell Spheres^a^

	FF5	FF15	FF25
core diameter [nm]	3.2(2)	15.4(2)	21.3(2)
shell thickness [nm]	6.4(2)	4.9(1)	6.9(1)
core SLD [10^–4^ nm^–2^]	6.9	6.9	6.9
shell SLD [10^–4^ nm^–2^]	2.79(10)	2.40(15)	2.94(20)
power exponent	1.8(1)	2.2(1)	2.3(2)
distribution radius [%]	4.9	6.7	4.9
distribution shell thickness [%]	15	15	9.1

a The SLD of the cores was fixed, and the SLD of the solvent was allowed to vary in a tight range near 4.6 × 10^–4^ nm^–2^. For the definition of SLD see the Methods section.

Silicon (100) crystals (50 × 50 × 10 mm^3^, optically polished) were obtained from CrysTec^a^ (Germany) and used for the experiments. To provide high surface energy, one of the three wafers was chemically cleaned in freshly prepared piranha solution [50/50 (v/v)], H_2_SO_4_ (concentrated) and H_2_O_2_ (30% aqueous), resulting in a hydrophilic wetted surface with a contact angle of 6° for water. The other wafers were cleaned by the same method, and then a hydrophobic octadecyltrichlorosilane (OTS, contact angle 110°) monolayer or an APTES monolayer (contact angle 51°) was chemically grafted onto them. The grafts were obtained by vapor deposition where the substrates were exposed to the gaseous silanes for more than 6 h. The contact angles were obtained with fresh ultrapure water by using the sessile drop method.^27^

## Methods and Experiment

At a glancing angle to an interface, neutrons are either transmitted or reflected according to the changes in scattering potential, which is described by the scattering length density (SLD) ρ:^28^
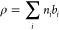
1Here, *n*_*i*_ is the number density for nuclei of isotope *i* and *b*_*i*_ is the bound nuclear coherent scattering length for neutrons for the respective nuclei. For all isotopes *b* is a unique and tabulated^29^ value describing the interaction potential between the neutron and the nuclei. As the wavelength of the neutron is much larger than the extension of the nuclei, the interaction potential can be described by a delta function and *b* is a single number. By use of this interaction potential, the refractive index *n*_r_ for a given material is calculated for neutrons from the SLD and the wavelength λ:
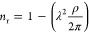
2Note that as the interaction potential between the neutron and the nuclei is small, the refractive index of neutrons for all materials is very close to one. In addition, the interaction potential may be repulsive or attractive, and as a consequence of this the refractive index can be slightly larger or smaller than one. This is different from photons for which the refractive index can be related to the group velocity, which in matter is always smaller than the speed of light, *c*. From the refractive index and eq 2 the SLD profile across an interface can be extracted by the measurement of the reflected neutron beam intensity. Note that as the values of *b* are known, the number density of nuclei in a layer can be extracted from reflectivity or SANS experiments. This is different from ellipsometry with optical photons, where the dielectric function of the materials needs to be determined in separate measurements. Moreover, *b*_*i*_ is very different for H and D and actually negative for H and positive for D, which generates contrast between particular components in a sample. In the case of studying magnetic NP, the SLD of pure H_2_O is typically close to that of the particles’ shell material while that of pure D_2_O is close to magnetite (the magnetic core). Moreover, the SLD of D_2_O is large, resulting in high reflectivity. Considering this for our study, we have chosen a high fraction of D_2_O in the solvent to highlight the particle shells and have a high reflectivity signal.

**Figure 2:**
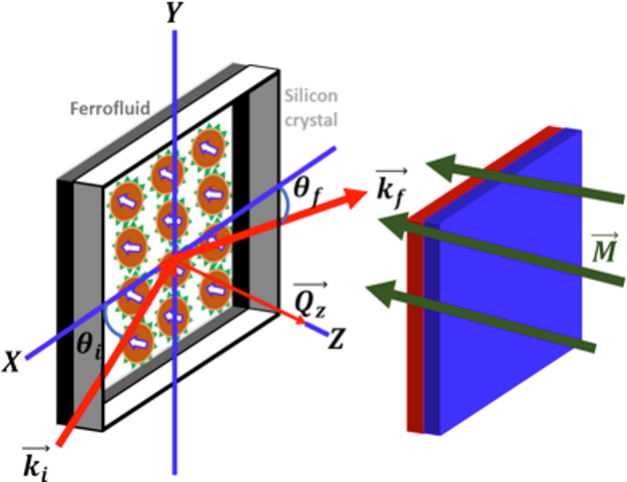
Sketch of the experimental setup showing the incident and reflected neutron beams (*k*_i_, *k*_f_), the nanoparticle assembly, and the perpendicular magnetic field applied by using permanent magnets. *Q*_*z*_ is the vector of momentum transfer.

The specular reflectivity, *R*(*Q*), is the ratio of the intensity of the reflected beam with respect to that of the incident beam for identical angle of the incident (θ_i_) and exiting beams (θ_f_). Note that other than for optical measurements these angles are defined with respect to the sample surface plane and are therefore small (see Figure 2). For this case, the momentum transfer *Q*_*z*_ = (4π/λ) sin(θ_i_) is perpendicular to the interface. Note that in this geometry neutron reflectometry is not sensitive to lateral density fluctuations along the interface. The SLD values extracted are average values over the coherence volume of the neutron beam, which is several micrometers in the plane of the interface. As a consequence in this study we only evaluate the layering of NPs but cannot access their local structure, which would require additional measurements of the so-called off-specular or grazing incidence small-angle scattering data.^30^ Similar to optics from the refractive index, a critical momentum transfer of total external reflection can be defined. For *Q* values exceeding this value reflectivity decreases following the Fresnel equation, proportional to *Q*^−4^. For rough surfaces an even steeper decrease is found. For more than one interface the specularly reflected intensities from the different interfaces interfere, providing information about the thickness, roughness, and composition the layered structure. Quantitative information can be extracted from model fits using the Parratt formalism.^31^

NR measurements were performed on the reflectometer MARIA^32,33^ at the outstation of the Jülich Center for Neutron Science (JCNS) at the Heinz Maier-Leibnitz Centre (MLZ, Garching, Germany) by using a vertical sample orientation. The assembled but empty sample cell was mounted on the instrument. Then first a measurement of the wafer against D_2_O was taken, which was then exchanged with the NP sample. The delay time until the measurement started is short (on the order of minutes) compared to the scanning time (2 h). The reflectivity data were collected with wavelength λ = 10 Å and λ = 5 Å for *Q* < 0.042 Å^–1^ for 0.035 Å^–1^ < *Q* < 0.2 Å^–1^, respectively, having a small overlapping region. One scan over the entire *Q* range took ∼2 h and was repeated after the respective waiting times. The wavelength spread was 10%, and this dominates the d*Q*/*Q* resolution at the used collimation setting. The scattering geometry and sample cell are described in Figure S2 of the Supporting Information. A collimated neutron beam penetrates the edge of the Si crystal and undergoes reflection at the silicon–liquid interface. A magnetic field of 100 and 250 mT was applied perpendicular to the Si interface by using permanent neodymium magnets. For the NR experiments the NPs, FF5, FF15, and FF25, were dissolved in a D_2_O/H_2_O mixture of 0.80/0.20, 0.78/0.22, and 0.78/0.22, respectively, with a concentration of 5 vol % Fe_3_O_4_.^c^ Approximately 1.5 mL of the FF sample was loaded into a wet cell^21^ sealed by a 2 mm thick (sample thickness) polytetrafluoroethylene (PTFE) gasket mounted between the coated silicon crystals and a polycarbonate plate. The size of the Si crystals was 5 × 5 × 1 cm^3^, and the thickness of the sample liquid was <1 mm to minimize magnetic field gradients. Note that as the absorption of neutrons in Si is small, no significant beam attenuation is observed, and as D_2_O has a larger SLD than Si, total external reflection is observed. The sample was injected into the sample cell directly after preparation (dissolving the NP powder).

**Figure 3:**
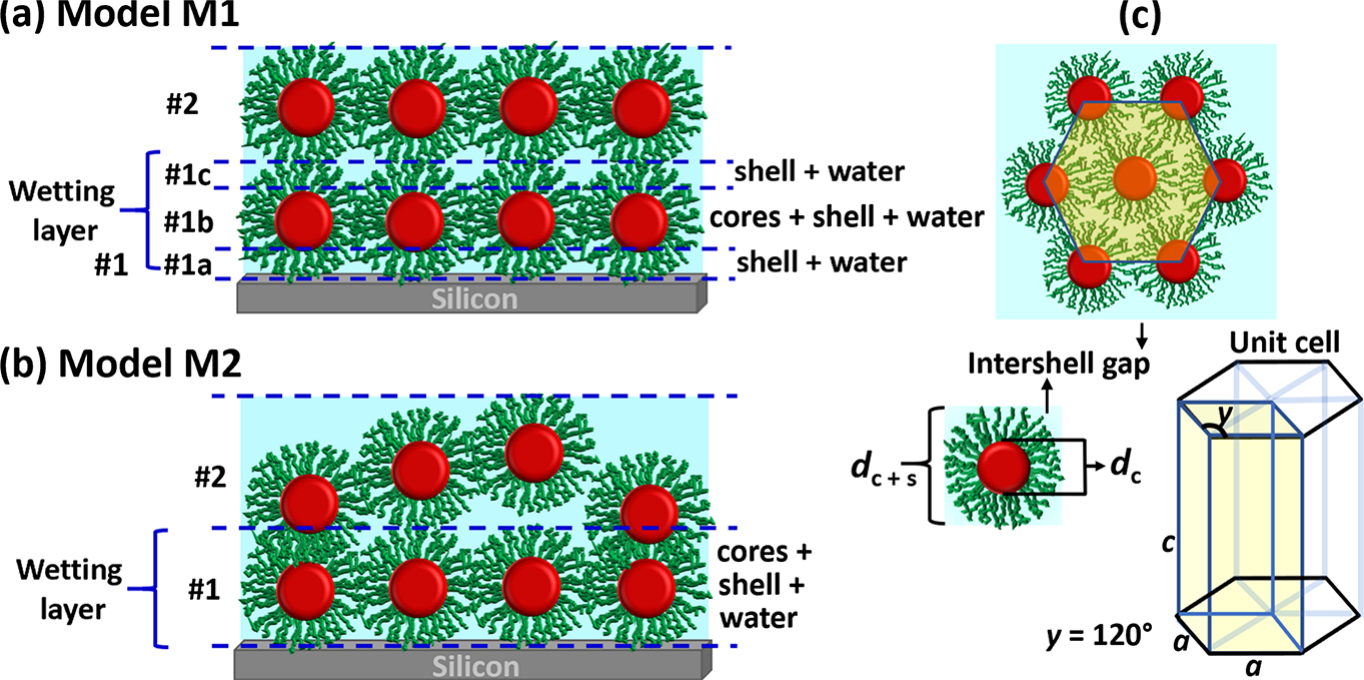
(a) Model for ordering of truncated hard-sphere core/shell particles in a wetting layer in a close-packed 6-fold arrangement. (b) Model for ordering of hard-sphere core/shell particles in a close-packed 6-fold arrangement. (c) Model schematics for visualizing the arrangement of core/shell particles in a hexagonally defined 6-fold (close-packed) arrangement.

The background-corrected reflectivity data were fitted employing the Parratt formalism^31^ by using the software package Refl1D.^34,35^ To fit the data, we considered two models (see Figure 3a,b). Model M1 (employed for samples FF15 and FF25) divides the first wetting layer (1) of particles into three sublayers. The first sublayer (1a) in contact with the substrate consists of mainly shell material. The second sublayer (1b) contains the magnetite cores as well as shell material and D_2_O/H_2_O between them. Finally, the third layer (1c) is composed of only shell material and water again. Because the volume fraction of cores with respect to shell material for sample FF5 is below 1%, the sublayers could not be resolved and model M2 (Figure 3b) is employed. Starting from the second wetting layer of particles (2) both models are identical, and no subdivision of layers is considered any more. For more details see the Supporting Information. To further analyze the data, we define criteria for close-packed (CP) layering by calculations of the SLD assuming fractional packing. Figure 3c (top) visualizes the structure of a close-packed layer of spherical particles with 6-fold symmetry. Assuming this structure and utilizing the core/shell diameters determined from SANS along with the bulk SLD values of the FF components, the SLD of a dense layer can be calculated for different water concentrations in the ligands and interstitial voids and compared to the fitting parameters extracted from the data. For this calculation, in the case of M1, the ligand shells above and below the tangent planes of spherical particles in the wetting layer were excluded and fitted as separate layers, while for M2 the SLDs of all components present in the layer, including core and shell material as well as solvent, are averaged over the total thickness of the layer. The thickness of this first wetting layer is found in good agreement with the NPs size for all experiments performed in this work. The SLD value for an ideal CP monolayer of NPs falls between the SLD values calculated assuming shell material or water in the interstitial voids.^21^ The two scenarios provide an upper and lower limit for a layer to be CP. Layers with a SLD outside this range are called loose packed (LP). Note that LP layers may be either layers of particles of lower density (surface coverage) or patches of densely packed particles separated by uncovered areas.^21^ As the coherence length of the neutrons along the surface is on the order of micrometers, these two scenarios cannot be distinguished. In addition, the thickness of layer (2) clearly exceeds the particle diameter in most cases and should be seen as a rough, not well organized, layer in those cases as indicated in Figure 3a (lower panel). The regions for CP layers are indicated by the gray areas in the SLD profiles in the Results section.

In all data sets a native SiO_2_ layer is assumed on the silicon substrate. This layer was fitted independently from measurements of the substrate in contact with D_2_O (not shown) and then kept fixed for the subsequent fits to the FF data. Note that since the actual APTES layer is very thin, the NR measurement is not sensitive to it due to the limited *Q*-range.

## Results

### Coating of the Solid Substrate

**Figure 4:**
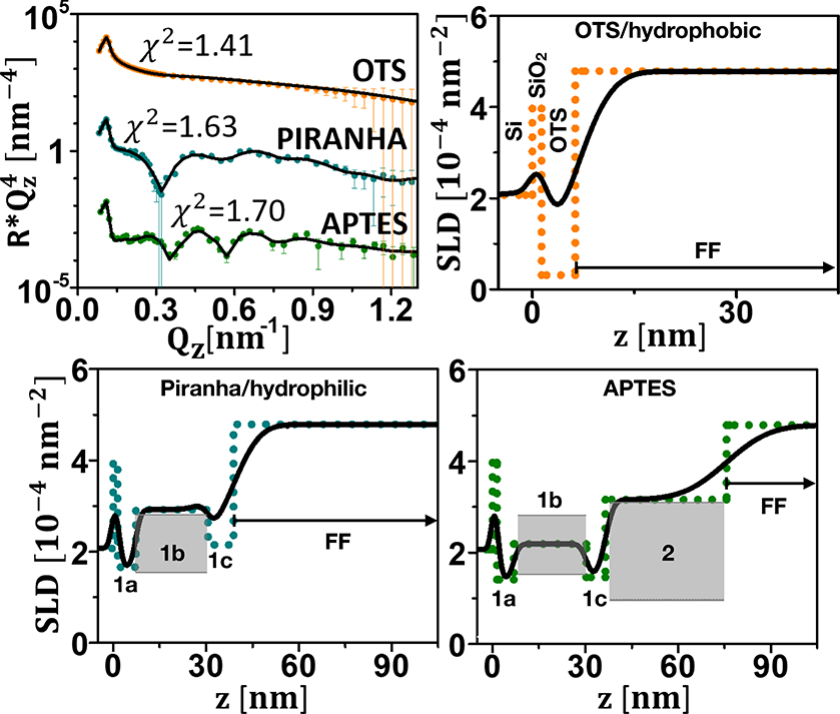
Upper left panel: NR () plotted as a function of *Q*_*z*_ for FF25 (5 vol %) measured against hydrophilic (piranha), hydrophobic (OTS), and APTES-coated Si. The solid lines represent fits to the data. Other panels: profile of nuclear SLD plotted as a function of distance from the Si (100) surface. Also included are the SLD values for the close-packed particle layers (gray areas). The dots show the SLD profile assuming zero roughness to aid identification of the distinctive layers, as defined in Figure 3. In the upper right panel the substrate, SiO_2_, and OTS layers are indicated as well. Error bars represent the statistical uncertainties propagated through the data normalization and with a one sigma confidence interval.

NR data along with the best fits and the corresponding SLD profiles are shown in Figure 4 for sample FF25 in contact with the silicon substrates with different coatings. Clearly, the particles do not self-assemble onto the surface coated with hydrophobic OTS. For the two other coatings, hydrophilic piranha and APTES, self-assembly is found. The first wetting layer can be subdivided in three distinct slabs (model M1). The NPs are at the edge of being CP with a relatively high water content of about 30%. In addition, a second wetting layer that is loosely packed and with a much higher content of solvent is formed.

### NP Size

**Figure 5:**
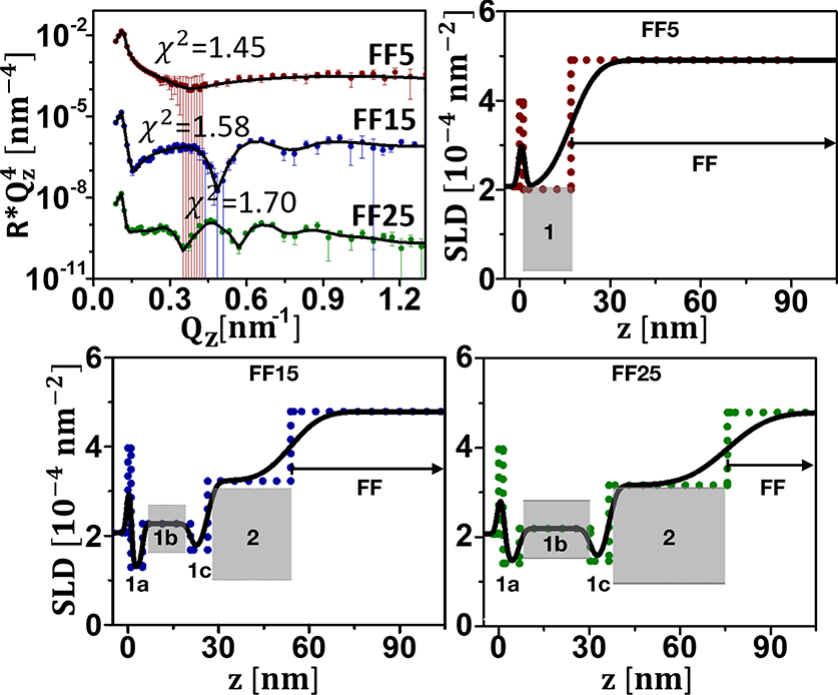
Upper left panel: NR () plotted as a function of *Q*_*z*_ for FF5, FF15, and FF25 (5 vol %) measured against APTES-coated Si. The solid lines represent fits to the data. Other panels: profile of nuclear SLD plotted as a function of distance from the Si (100) surface. Also included are the SLD values for the close-packed particle layers (gray areas). The dots show the SLD profile assuming zero roughness to aid identification of the distinctive layers, as defined in Figure 3. Error bars represent the statistical uncertainties propagated through the data normalization and with a one sigma confidence interval.

NR data, multiplied by , for the samples FF5, FF15, and FF25 (5 vol % solved in D_2_O/H_2_O) measured against APTES-coated Si are shown as a function of *Q*_*z*_ in Figure 5 (upper left panels). Data are taken with the samples in zero magnetic field. The best fits to the data with the corresponding χ^2^ (marked) are shown as solid lines. The corresponding SLD profiles are displayed in the other three panels. For FF5, the wetting layer at the SiO_2_ interface is a particle monolayer consisting of a mixture of shell material (ligands attached to the NPs and in the interstitial regions between the NPs), excess surfactant, core material, and water. No additional NP layers can be differentiated between this slab and the bulk liquid for FF5. For samples FF15 and FF25, the first sublayer of the wetting layer (model M1) in contact with the SiO_2_ consists of shell material, excess surfactant, and water. The center of the wetting layer can be identified and contains the particle cores with shell material in-between as well as some water. This layer is followed by ligands. The three layers defined in M1 (Figure 3a) form a CP wetting layer. For these two samples an additional LP layer, with a water content of almost 50%, is found between the wetting layer and the bulk liquid.

### Magnetic Field

**Figure 6:**
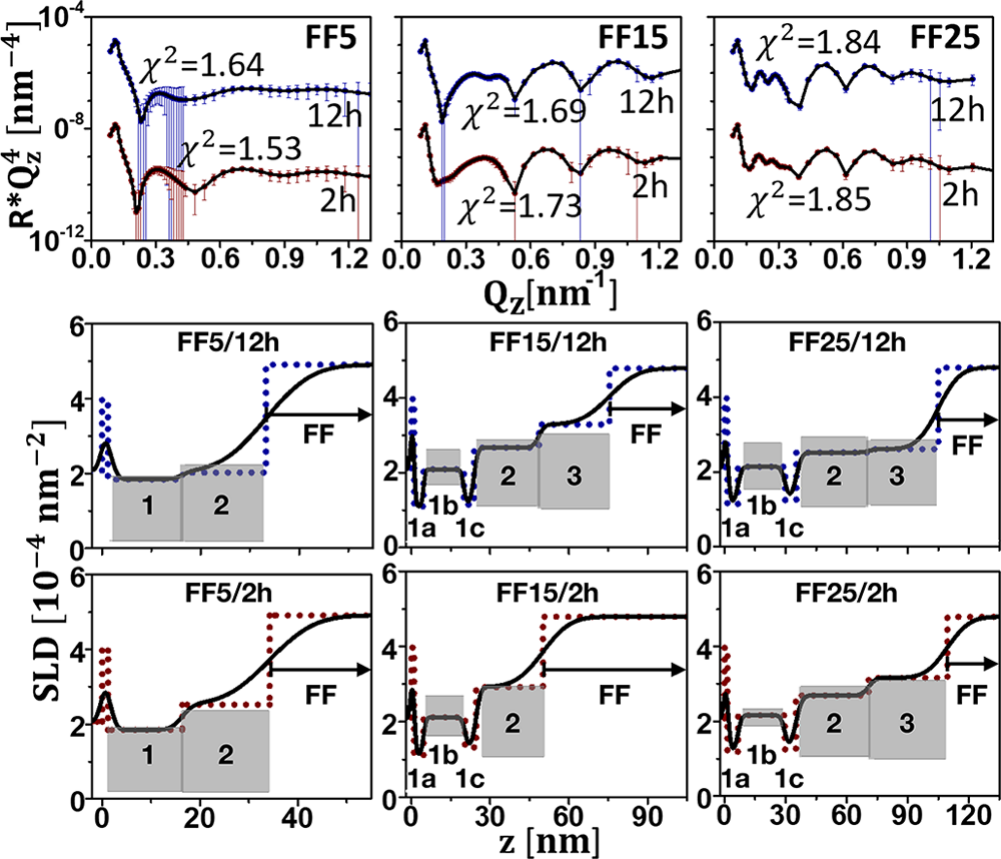
Upper left panel: NR () plotted as a function of *Q*_*z*_ for NP sizes of 5, 15, and 25 nm and a magnetic field of 100 mT applied out-of-plane and measured with the samples in contact to an APTES substrate. The solid lines represent fits to the data. Other panels: profile of nuclear SLD plotted as a function of distance from the Si (100) surface. Also included are the SLD values for the close-packed particle layers (gray areas). The dots show the SLD profile assuming zero roughness to aid identification of the distinctive layers, as defined in Figure 3. Error bars represent the statistical uncertainties propagated through the data normalization and with a one sigma confidence interval.

Figure 6 shows data taken with all three APTES samples and a magnetic field of 100 mT applied out-of-plane for 2 and 12 h. After 2 h under a magnetic field of 100 mT additional particles wet the surface for all samples. This observation is in good agreement with previous studies.^19^ In sample FF5 a continuous densification of a second wetting layer with a water content that decreases with time is found. In both FF15 and FF25, the initial LP second layers become CP after 2 h with a high water content of 40% and 35%, respectively. For sample FF25 even an additional third LP layer water content of 47% is reported. Applying the magnetic field for longer times, after 12 h, results in the densification of this third layer, which becomes CP (water content 32.5%). After this time with 100 mT applied also for sample FF15 a third LP layer with high water content is reported (water content 50%). Note that as we use permanent magnets to generate the magnetic field inhomogeneities cannot be excluded. These may lead to additional self-assembly, as observed in ref (36), but with the same trend of more pronounced assembly for the larger particles.

## Discussion

**Figure 7:**
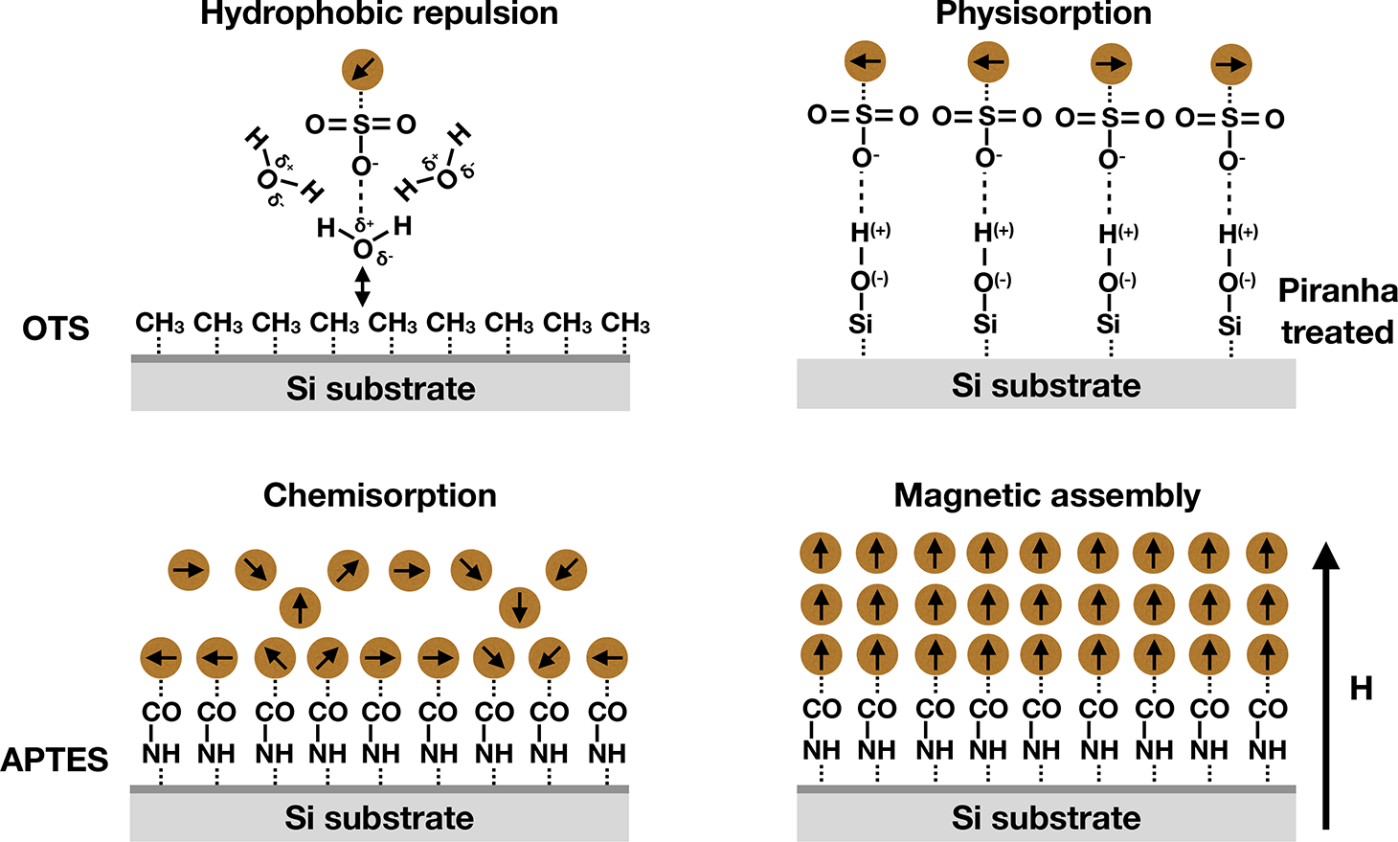
Magnetic self-assembly of NP at Si substrates with different surface termination. For the case of chemisorption, a dense wetting layer of magnetic particles is formed, which allows the assembly of adjacent layers via the magnetic dipolar interaction.

To understand the self-assembly process of magnetic NPs at solid substrates, the relevant interactions have to be considered. In this study we have a focus on the termination of the solid substrate (chemical and physical absorption) as well as magnetic dipolar interaction, which is the only longer range interaction present in the samples. From our study of different surface terminations of the silicon substrates it is clear that only for the appropriate coating magnetic NPs may assemble. This can be well understood since the particles reach the surface in a random manner and only stick to it if short-range attractive interactions exist. The NHS conjugated NPs chemically couple with APTES by a strong bonding. Piranha-treated hydrophilic substrates present hydroxy (−OH) terminations^22^ to the −COOH polarities of the NPs, resulting in a hydrogen bond formation between the two. The bond is strong but weaker than the −CONH bond obtained with APTES.^23^ The OTS coating is a methyl (CH_3_)-terminated alkylsilane.^37^ Note that the ligands charge stabilize the NP in water and are strongly hydrated (see Figure 7, upper left panel). As such, they can be treated as hydrophilic and show no affinity to the OTS coating, which is strongly hydrophobic. Figure 7 summarizes the formation of the first wetting layer by either physisorption (piranha-treated surface) or chemisorption (APTES coating), panels upper right and bottom left, respectively.

The particles in sample FF5 are superparamagnetic (SPM) at room temperature. The magnetic anisotropy energy of these NPs is smaller than the thermal energy, and thus no magnetic moment can be stabilized without the application of an external magnetic field^38^ (Neel relaxation). The critical size (SPM limit) for ferrimagnetic Fe_3_O_4_ NPs is below or close to 15 nm.^39^ This explains why only one loose-packed wetting layer is observed for sample FF5 in contact with APTES. Note that the volume fraction of magnetic core material in these particles is below 1%, and even at dense packing, the distance between cores is relatively large and no induced moments between NPs can be expected. As a result, no sublayers can be identified in the SLD profiles fitted to the NR. Moreover, even for the case of two wetting layers a clear distinction between them remains challenging, and they rather manifest in one thick region of low SLD of increasing hydration for distances further from the substrate (see Figure 5, upper right panel, and Figure 6, lower left panels). The NPs in samples FF15 and FF25 are slightly or clearly above the SPM limit for Fe_3_O_4_. Therefore, the individual NPs are ferrimagnetic and single domain with a large remanence and an uniaxial anisotropy axis.^21^ However, when dissolved in water, the whole particle may rotate (Brownian relaxation). Altogether, all samples, NP dissolved in water, investigated in this work show a magnetic behavior with no remanence and high susceptibility at small externally applied magnetic fields. If now adsorbed at the silicon substrates, the NP cores come closer to each other and may interact via magnetic dipolar forces and form domains.^19^ The larger particles may rotate with their uniaxial anisotropy axis along the magnetic field lines. The magnetization in the domains is expected to be in the plane of the substrate to facilitate the formation of long end-to-end dipole chains, as the result of the attractive force between adjacent particles. However, out-of-plane stray fields exist at the domain walls. The stray fields decrease with increasing distance from the substrate, and the NPs in solution experience a force due to their magnetic dipole moment and the field gradient. As a result, further wetting layers, depending on the magnetization of the NP, may assemble at the interface (see Figure 7, bottom left panel). As the magnetic moment of the larger NPs is greater, this effect gets more pronounced with increasing size of the NPs.

If an out-of-plane magnetic field is applied, the situation changes. Because all samples have a large susceptibility, the magnetization of the NP will align with the external field and point out-of-plane as well. Moreover, only part of the substrate is covered with magnetic cores, since either shell material or water is found in between the NPs even in the case of dense packing. In total, this results in field gradients and out-of-plane magnetic fields, which attract NPs from solution. If present, the magnetic particles further enhance field gradients present from the permanent magnet mounted above the silicon crystal (see the Supporting Information). Whenever a NP reaches the wetting layer, it gets stabilized above the particles in the first wetting layer to have a head to tail magnetic moment, as shown in Figure 7 (bottom right). As a result, we observe additional wetting layers for all three samples developing with time in an out-of-plane magnetic field. For longer times as well as larger magnetic fields the layering becomes more pronounced (see the Supporting Information), as the magnetic interaction has to overcome the steric and electrostatic repulsion between the NPs. Moreover, we do not see large effects of the NP concentration (see the Supporting Information), which is in line with the assumption that the NP get stuck once they are chemically anchored at the interface as well as with the fact that on long length scales the magnetic dipolar interaction dominates.

To highlight more details on the dependency of the assembly of the NP on their size and moment, Table 2 summarizes the wetting layers formed at the APTES substrate for different particles sizes and applied magnetic fields. The water content was calculated, along with whether a layer can be identified as close-packed, from a comparison of the SLD to the possible dense packing regions indicated in gray in the SLD profile figures in the Results section. At the upper limit of SLD all interstitial voids are filled by water, and the lower limit indicates only core material and ligands in the layer. Clearly and as expected from the discussions of the magnetic moment of the particles, more dense packed layers are formed for the particles of larger size since they have larger moments and a larger volume fraction of cores in dense layers. At the same time, a lower water content is found in the layers. Under the application of an out-of-plane field in all samples, additional layers assemble, and those already existing at zero field become more dense. This observation continues over at least 12 h, which is the longest time investigated in this study. After this time, for sample FF15 a third loose-packed layer is observed, which became close-packed for sample FF25. The presence of a third particle layer on-top of the wetting layer is a new observation that contrasts with results from our previous studies under in-plane magnetic field.^19,20^

**Table 2:** Packing Density and Water Content Summarized for All Layers^a^

		packing			water content [%]		
L	H	1	2	3	1/1b	2	3
FF5	0	LP			38		
	2 h	CP	LP		35	49	
	12 h	CP	LP		35	38	
FF5*	0	LP			45		
	2 h	LP			40		
	12 h	LP			40		
	24 h**	CP	LP		36	39	
FF15	0 h	CP	LP/CP		14***	48	
	2 h	CP	LP/CP		11***	40	
	12 h	CP	CP	LP	9***	38	50
FF25	0	CP	LP/CP		14***	47	
	2 h	CP	CP	CP/LP	13***	35	47
	12 h	CP	CP	CP	6***	32	33

a Row L identifies the wetting layers 1, 2, and 3. Column H states the time of the out-of-plane magnetic field of 100 mT applied to the sample. The value indicated with ∗∗ was at a field of 250 mT (data not shown in the main text; see the Supporting Information). Samples indicated by ∗ are dilute solutions of 0.5 vol % (data not shown in the main text, see the Supporting Information). Water concentrations indicated by ∗∗∗ for the first wetting layer of samples FF15 and FF25 are calculated for sublayer 1b (see Figure 3a).

## Conclusion

NR measurements were reported for magnetite nanoparticles dissolved in water with nominal size of 5, 15, and 25 nm (FF5, FF15, and FF25) at a concentration of 5 vol %, under zero field, after 2 and 12 h of applying an out-of-plane magnetic field of 100 mT, adjacent to differently functionalized silicon substrates. The reflectivity data reveal that a wetting layer of magnetic NPs only forms at a silicon interface if the particles are either physisorbed or chemisorbed. The densest layers are found for the stronger chemical binding. Once formed, this first wetting layer results in magnetic stray fields attracting further particles, which may form a second layer. This layer is only observed for NPs which are inherently ferrimagnetic and rotate via Brownian motion to align their anisotropy axis with the local magnetic field. Generally, larger NPs with larger moments show better layering. Once an out-of-plane magnetic field is applied, additional layers form and the existing ones become denser packed. This densification continues over the whole time of the investigation of up to 12 h.

Our results show that careful control of the surface chemistry of a substrate can be used to create seed layers of magnetic particles of well-defined structures. During the self-assembly process, the particle size and magnetic moment (dipolar interaction) are the key factors for the formation of dense layers. Application of a magnetic field promotes further particle layering. Our results provide a path forward for controlling and tuning these self-assembled structures for device applications.

## Supporting Information Available

Model calculations of the SLDs assuming voids being filled with ligands or solvent for all layers; the resulting values are tabulated, and NR data with fits and resulting SLD profiles are shown for a dilute sample of FF5 as well as for larger magnetic fields applied (PDF)

## Footnotes

a Certain commercial equipment, instruments, or materials (or suppliers, or software, etc.) are identified in this paper to foster understanding. Such identification does not imply recommendation or endorsement by the National Institute of Standards and Technology, nor does it imply that the materials or equipment identified are necessarily the best available for the purpose.

b Values in parentheses are uncertainties counting from the last digit, thus 4.1(5) representing 4.1 ± 0.5.

c Sample FF5 was measured at a dilute concentration of 0.5% in addition to the 5% concentration. The data are included in the Supporting Information, and the results are summarized in Table 2.
