# Supplementary material for: Magnetic Particle Self-Assembly at Functionalized Interfaces
Source: Langmuir. 2021 Apr 2;37(14):4064–71. doi: 10.1021/acs.langmuir.0c03235 (PMC8154863; doi:10.1021/acs.langmuir.0c03235)
Supplement: Supplementary file 1 — la0c03235_si_001.pdf [file la0c03235_si_001.pdf]

Supplementary information

Magnetic particle self-assembly at functionalized interfaces

Apurve Saini,[†] Katharina Theis-Bröhl,[‡] Alexandros Koutsioubas,[¶] Kathryn L. Krycka,[§] Julie A. Borchers,[§] and Max Wolff[*,†]

[†]Department for Physics and Astronomy, Uppsala University, Sweden
[‡]University of Applied Sciences Bremerhaven, Germany
[¶]Jülich Centre for Neutron Science JCNS at Heinz Maier-Leibnitz Zentrum (MLZ) Forschungszentrum Jülich GmbH, Lichtenbergstraße 1, 85748 Garching, Germany.
[§]NIST Center for Neutron Research, Gaithersburg, 20899-6102, USA

This supplementary material contains 7 pages and 8 figures.

1. Functionlisation of the magnetic nano-particles

The purchased NPs are functionalized by NHS (see figure S1). The NPs are each comprised of magnetic core (Fe_3O_4), oleic acid group (R), and the N-Hydroxysuccinimide group (NHS). The NHS group is amphiphilic; therefore, its hydrophilic end protrudes out in water and its hydrophobic end interacts with the hydrophobic end of the oleic acid group which is -CH3. The particles are stabilized in water by steric interactions (hydrophobic-hydrophobic) between the oleic acid group and the NHS moiety. In addition, repulsion among the particles is achieved by the negative charge on the SO_3 group, which is expected to be screened over the Debye length. As a consequence, on longer length scales the interactions among particles are dominated by the magnetic dipolar interaction.

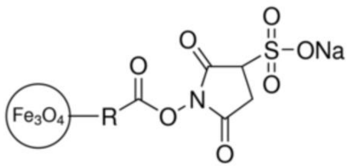

Fig S1: NHS stabilized particle.

These particles were dispersed in water and within the next few minutes injected into the previously mounted neutron reflectivity cell. The particles when dispersed in water were stable and did not agglomerate as concluded from DLS measurements after almost a month. Also, we did not see agglomeration in the SANS measurements, which took over several hours. Agglomeration on the time scales of our experiments can be neglected.

2. Characterisation of the magnetic nano-particles

A more detailed summary of the NP characterization is provided in Ref. [21], including electron microscopy and x-ray diffraction data. To provide experimental data on the key parameters we show the results of magnetization measurements (SQUID) and the SANS data, summarized in Table 1, in Fig. S2 (adapted from Ref. 21).

Fig. S2 (right panel) shows SANS data together with fits, which assume a power exponent together with polydispersed core/shell spherical NPs for each sample. Including the power law is needed to describe the low-Q upturn frequently seen in magnetic nanoparticle colloids [S1, S2]. It likely originates from excess surfactant since the power-law scattering contrast is pronounced in the D_2O (rather than the H_2O) solvent, which has a pronounced contrast with the surfactant. In addition, only minimal evidence of a correlation peak associated with long-range inter-particle ordering is found. We note that low-Q scattering associated with excess surfactant has been described by others on related systems [S3]. The SLD values for the shell material are larger than that of bulk shell material (0.16×10^{-4} nm^{-2}) due to the presence of water in the shell, which can be estimated from the fitted SLDs for the shells (see Table 1) and those of the solvent (4.6×10^{-4} nm^{-2}) and bulk shell material. We find approximately 59 %, 50 % and 63 % of water in the shells for samples FF5, FF15 and FF25, respectively. For more details we refer to Ref. 21.

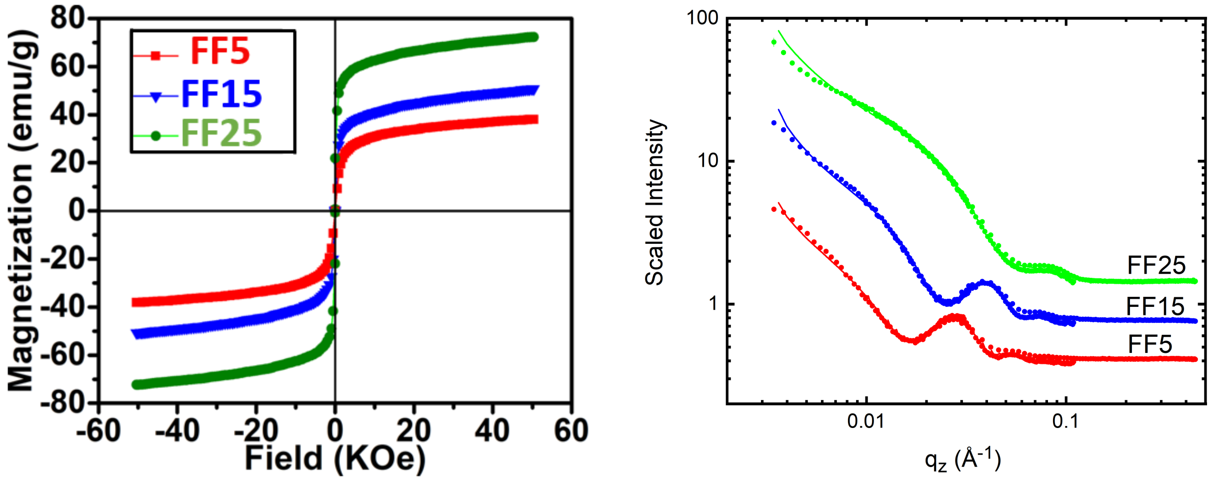

Fig. S2: The panel to the left shows hysteresis loops for iron oxide nanocrystals FF5 (red), FF15 (blue) and FF25 (green) at 300 K as extracted from SQUID measurements of the dried powder. The panel to the right shows SANS data for samples FF5 (red symbols), FF15 (blue symbols) and FF25 (green sym- bols) and fits to the data (solid lines). Data for FF5 and FF15 are scaled by a factor of four and two for better visibility, respectively. Figure adapted from Ref. 21.

3. Summary of packing and compositions

Sketches of the packing densities and concentrations of the constituents as calculated from the fits to the neutron reflectivity data and calculations assuming a hexagonal dense packing are shown in Fig. S3.

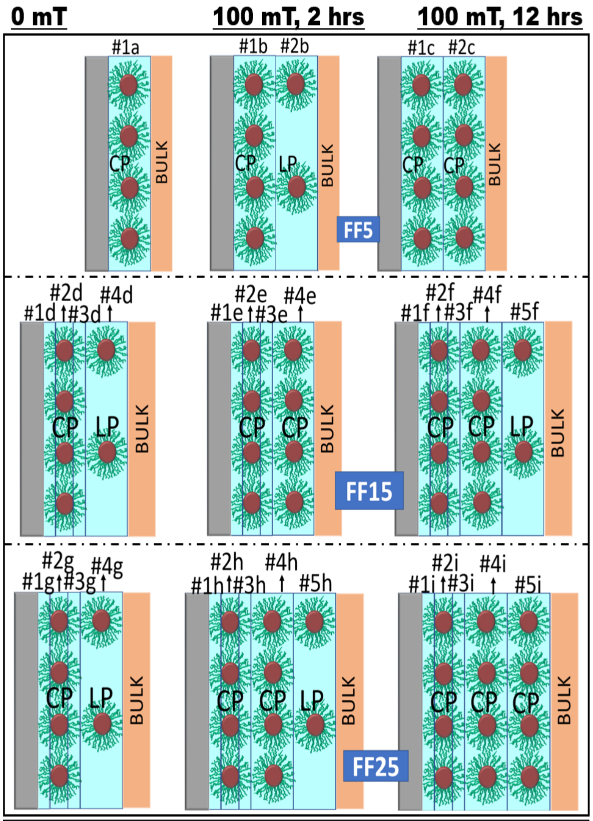

FF5

#1a	#1b	#2b	#1c	#2c
C_c : 0.50<br>C_s : 61.26<br>C_w: 38.23	C_c : 0.52<br>C_s : 64.64<br>C_w: 34.84	C_c : 0.45<br>C_s : 50.50<br>C_w: 49.05	C_c : 0.52<br>C_s : 64.85<br>C_w: 34.62	C_c : 0.47<br>C_s : 61.04<br>C_w: 38.49

(%)
C_c : Conc. core
C_s : Conc. shell
C_w: Conc. water

FF15

#1d	#2d	#3d	#4d	#1e	#2e	#3e
C_s : 76.41<br>C_w: 23.59	C_c : 21.69<br>C_s : 64.36<br>C_w: 13.95	C_s : 68.27<br>C_w: 31.73	C_c : 12.53<br>C_s : 39.43<br>C_w: 48.04	C_s : 79.96<br>C_w: 20.04	C_c : 21.76<br>C_s : 67.63<br>C_w: 10.61	C_s : 75.78<br>C_w: 24.22

#4e	#1f	#2f	#3f	#4f	#5f
C_c : 13.55<br>C_s : 46.59<br>C_w: 39.86	C_s : 80.38<br>C_w: 19.62	C_c : 22.44<br>C_s : 68.59<br>C_w: 8.97	C_s : 79.33<br>C_w: 20.67	C_c : 13.95<br>C_s : 48.50<br>C_w: 37.55	C_c : 12.82<br>C_s : 36.76<br>C_w: 50.42

FF25

#1g	#2g	#3g	#4g	#1h	#2h	#3h
C_s : 73.9<br>C_w: 26.10	C_c : 20.31<br>C_s : 65.45<br>C_w: 14.24	C_s : 72.86<br>C_w: 27.14	C_c : 12.17<br>C_s : 40.78<br>C_w: 47.06	C_s : 78.5<br>C_w: 21.50	C_c : 25.29<br>C_s : 68.38<br>C_w: 6.33	C_s : 77.24<br>C_w: 22.76

#4h	#5h	#1i	#2i	#3i	#4i	#5i
C_c : 13.36<br>C_s : 51.47<br>C_w: 35.17	C_c : 12.28<br>C_s : 40.83<br>C_w: 46.89	C_s : 79.54<br>C_w: 20.46	C_c: 20.57<br>C_s : 66.66<br>C_w: 12.77	C_s : 77.24<br>C_w: 22.76	C_c : 13.36<br>C_s : 55.14<br>C_w: 31.50	C_c : 13.99<br>C_s : 53.49<br>C_w: 32.52

Fig. S3. Schematics for the ordering of magnetic NPs (5 vol. %) close to APTES coated Si under different conditions of applied magnetic fields (top panels 5 nm particles, middle panel 15 nm and lower panels 25 nm) The tables at the bottom summarize the respective concentrations in each slab.

4. Sample cell for neutron reflectometry

For the NR experiments, a solid-liquid cell with a loading capacity of 2.5 ml was designed. Fig. S4 shows a sketch of the solid-liquid cell. The wet cell comprises of a 2 mm thick polytetrafluoroethylene (PTFE) gasket (with injection and outlet ports) used for containing and sealing the liquid sample between the silicon crystal (50×50×10 mm) and the polycarbonate base. The crystal and the base plate were clamped together with an aluminum frame. The cell was further supported on a frame for mounting on the neutron instrument, see Fig. S5(a).
For applying an out-of-plane magnetic field, a neodymium magnet (40×40×15) mm was placed behind the silicon substrate (10 mm distance from the sample), Fig. S5(b). The total thickness of the liquid sample was less than 1 mm and the field at the sample position was measured using a Hall probe. Given the small thickness of the sample field gradients are samll, if present at all.
During the neutron experiments the maximum beam footprint of the silicon wafer was 45 mm and decreasing with increasing incident beam angle to minimize edge effects.

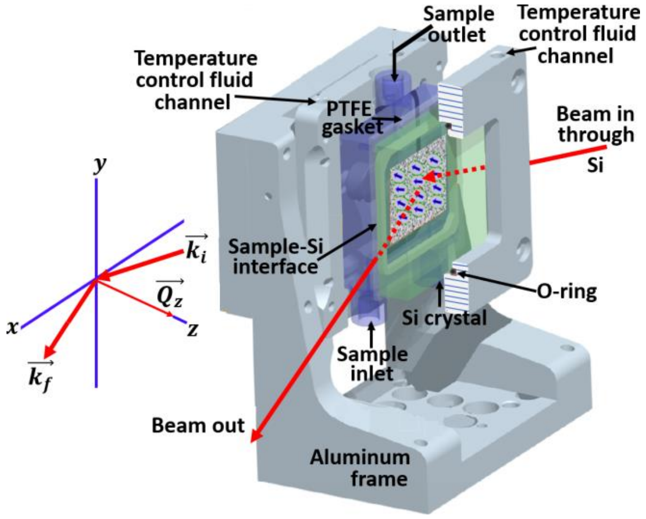

Fig. S4. Sketch of the solid-liquid cell with cutaway view of the holder for the substrate, liquid and gasket. The scattering geometry is shown on the left-hand side.

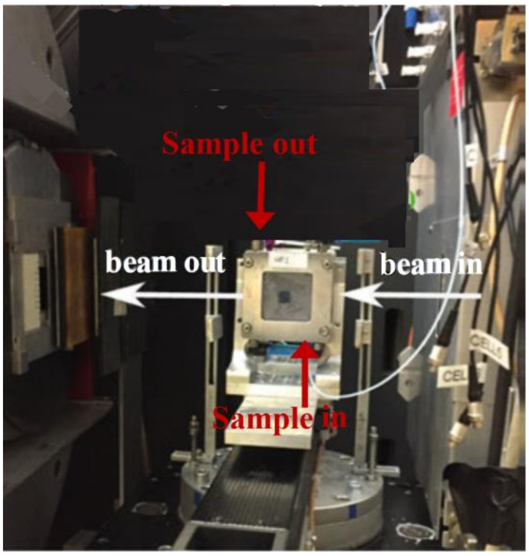

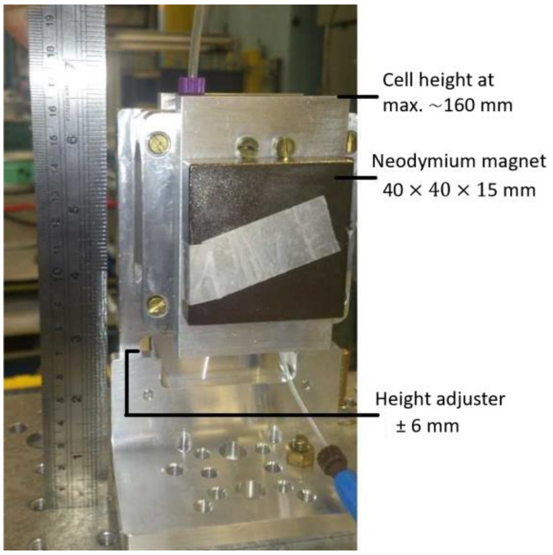

Fig. S5 (a) Solid-liquid cell (front face-silicon) mounted on instrument MARIA. **(b)** Cell with a permanent magnet attached behind the silicon crystal.

5. Dilute sample and magnetic field of 250 mT

Figure S6 shows neutron reflectivity curves plotted versus Q_z and normalized to Q^{-4} for a more dilute (0.5 vol%) solution of FF5 in contact to a APTES coated substrates for applied out-of-plane magnetic fields of up to 250 mT. The corresponding SLD profiles are shown in the figure as well. It turns out that only when a field of 250 mT is applied the first wetting layer becomes close packed. The layer structures and compositions are sketched in Figure S7 and S8.

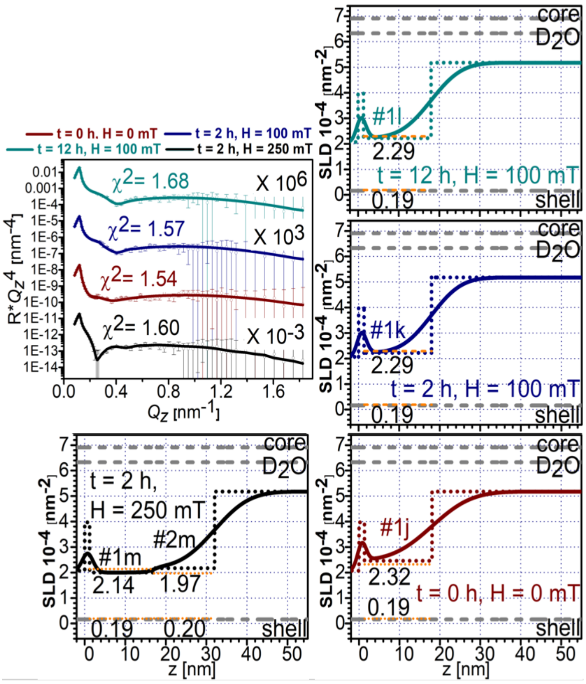

Fig. S6. NR data for FF5 (0.5 vol. %) taken without (red symbols) and with a magnetic field of 100 mT perpendicular to the Si (100) for 2 hrs (blue symbols) and after 12 hrs (teal symbols). Data for the sample under a field of 250 mT applied for 2 hrs (black symbols) is shown as well. (left) Plot of R. Q_z^4 as function of Q_z. The solid lines represent fits to the data. (right) Profile of nuclear SLD plotted as function of distance from the Si (100) surface. Also included are the SLD values for the magnetite core, water and shell material (gray dashed lines), as well as for the close-packed particle ordering within the slabs (orange dashed lines).

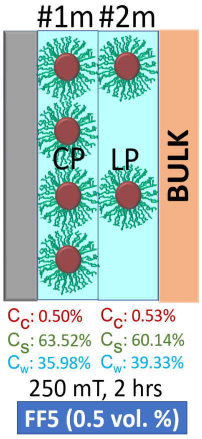

Fig. S7. Schematic layer model for the ordering of FF5 (0.5 vol. %) close to APTES coated Si under 250 mT applied for 2 hrs. Also, shown are the calculated compositions for each slab.

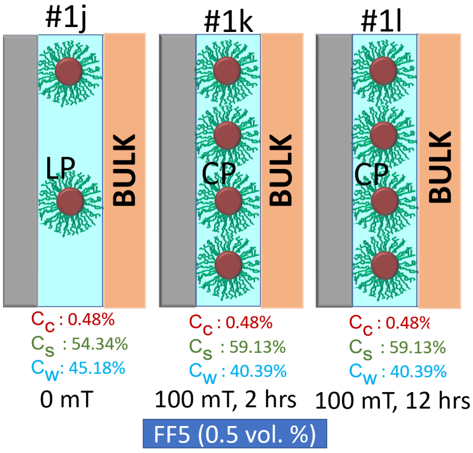

Fig. S8. Schematic layer models for the ordering of FF5 (0.5 vol. %) close to APTES coated Si under different conditions of applied magnetic fields. Also, shown are the calculated compositions for each slab.

References:
S1 Dennis, C. L.; Jackson, A. J.; Borchers, J. A.; Hoopes, P. J.; Strawbridge, R.; Foreman, A. R.; van Lierop, J.; GrÃijttner, C.; Ivkov, R., Nearly complete regression of tumors via collective behavior of magnetic nanoparticles in hyperthermia, *Nanotechnology* 2009, *20*, 395103.
S2 Bender, P.; Wetterskog, E.; Honecker, D.; Fock, J.; Frandsen, C.; Moerland, C.; Bog- art, L. K.; Posth, O.; Szczerba, W.; Gavilán, H.; Costo, R.; Fernández-Díaz, M. T.; González- Alonso, D.; Fernández Barquín, L.; Johansson, C., Dipolar-coupled moment correlations in clusters of magnetic nanoparticles, *Phys. Rev. B* 2018, *98*, 224420.
S3 Petrenko, V. I.; Avdeev, M. V.; Bulavin, L. A.; Almasy, L.; A., G. N.; Aksenov, V. L., Effect of surfactant excess on the stability of low-polarity ferrofluids probed by small-angle neutron scattering, *Crystallography Reports* 2016, *61*, 121.
